# Impact of Heating Temperature and Fatty Acid Type on the Formation of Lipid Oxidation Products During Thermal Processing

**DOI:** 10.3389/fnut.2022.913297

**Published:** 2022-06-02

**Authors:** Yuan Zhuang, Jun Dong, Xiaomei He, Junping Wang, Changmo Li, Lu Dong, Yan Zhang, Xiaofei Zhou, Hongxun Wang, Yang Yi, Shuo Wang

**Affiliations:** ^1^State Key Laboratory of Food Nutrition and Safety, Key Laboratory of Food Nutrition and Safety, Ministry of Education of China, Tianjin University of Science and Technology, Tianjin, China; ^2^Tianjin Key Laboratory of Food Science and Health, School of Medicine, Nankai University, Tianjin, China; ^3^School of Life Science and Technology, Wuhan Polytechnic University, Wuhan, China; ^4^School of Food Science and Engineering, Wuhan Polytechnic University, Wuhan, China

**Keywords:** thermal treatment, edible oil, fatty acid, lipid oxidation products, α, β-unsaturated aldehydes

## Abstract

Thermal treatment of lipids rich in fatty acids contributes to the formation of lipid oxidation products (LOPs), which have potentially harmful effects on human health. This study included soybean oil (SO), palm oil (PO), olive oil (OO), and lard oil (LO) as the research objects, with an aim to investigate the impact of heating temperature and fatty acid type on the generation of LOPs (α-dicarbonyl compounds, malondialdehyde (MDA), α,β-unsaturated aldehydes, and 16 volatile aldehydes). Results showed that LOPs increased significantly (*p* < 0.05) with the increase in temperature (100 ~ 200°C). Furthermore, the amount of 2,3-butanedione (159.53 μg/g), MDA (3.15 μg/g), 4-hydroxy-hexenal (3.03 μg/g), 2-butenal (292.18%), 2-pentenal (102.26%), hexanal (898.72%), and 2,4-heptadienal (E, E) (2182.05%) were more at 200°C in SO rich in polyunsaturated fatty acids (PUFAs) than other oils. Results from heat map analysis indicated that the 2, 4-heptadienal, and glyoxal related to the myristic acid of oil. Moreover, the MDA was in close association with PUFAs. Based on the effect of temperature and fatty acid type on the generation of LOPs, this study could serve as a control method to reduce harmful LOPs.

## Introduction

The thermal treatment is a common cooking method, for instance, steaming, boiling, stir-frying, frying, and roasting. Oil is a necessity for thermal treatment and an important part of people's diets that comprises a number of nutrients, such as essential fatty acids (1, 2). The temperature of these methods ranges from 100°C to 200°C, which is the most significant period for oil to provide good flavor and golden coloration. However, the oil can generate potentially hazardous substances like lipid oxidation products (LOPs) due to hydrolysis and oxidation of oil exposed to oxygen and moisture under high temperatures (3, 4). During thermal treatment of oil, oxidation of unsaturated fatty acids (UFAs) leads to the generation of various radicals, including ∙CH3, ∙CO, and ∙CHO, which could subsequently generate malondialdehyde (MDA), α-dicarbonyl compounds (α-DCs) such as glyoxal (GO), methylglyoxal (MGO), and 2,3-butanedione (2,3-BD), α,β-unsaturated aldehydes such as 4-hydroxy-2-hexenal (4-HHE) and 4-hydroxy-2-nonenal (4-HNE), and volatile oxidation products such as 2-butenal and pentanal (5, 6). LOPs could cause deleterious health effects in humans in animal- and cell-based toxicity studies such as elevated plasma total cholesterol and low-density lipoprotein cholesterol levels (7, 8) as long time exposure to these toxic compounds, which could binding with proteins and DNA (9, 10). Hence, understanding the formation of LOPs and controlling the LOPs by processing method and food matrix during oil thermal treatment have considerable practical relevance as well as theoretical interest.

The factors affecting the oxidation of edible oil have been studied, among which the predominant factors that influence the formation of LOPs were processing method and precursor (11). Some recent reports showed that the total amount of GO, MGO, and 2,3-BD formed 55- and 15-fold in heated butter and margarine (from 100 to 200°C), and the volatiles were increased with the temperature and constant with time in olive oils (5, 12). The content of 4-HNE was increased in the sunflower oil (185°C for 5 h) rich in linoleic acid (LA) and was more susceptible to decomposition (13). The close relationship between the UFAs and their corresponding volatile aldehydes was also revealed by some studies (14, 15). However, only limited LOPs types had been obtained during lipid oxidation under thermal temperatures. Hence, a wider variety of LOPs were affected concurrently by temperature and fatty acid needs further comprehensive investigation.

Compared to the previous study, this work in the full scale measured the secondary lipid oxidation products during thermal treatment and analyzed the relationship between fatty acids and extensive lipid oxidation products. Furthermore, the different thermal temperatures simulated the different food cooking methods to explore the different food processing-temperature affecting the formation of lipid oxidation products in different saturation oils. The purpose of this research was to investigate the formation of LOPs (α-DCs, MDA, α, β-unsaturated aldehydes, and 16 kinds of volatile aldehydes) in soybean oil (SO), palm oil (PO), olive oil (OO), and lard oil (LO) and elucidate the impact of fatty acids (myristic acid, palmitic acid, palmitoleic acid, stearic acid, oleic acid, linoleic acid, and linolenic acid) and temperatures (100, 120, 150, 180 and 200°C) on LOPs. Based on the generation of LOPs, this study could serve as a control method to reduce harmful LOPs. We could also evaluate the nutritional value of oil-type food processed through different methods.

## Materials and Methods

### Materials

Methanol, acetonitrile, and n-hexane of chromatographic grade were bought from Merck (Damstadt, Germany). 2,4-Dinitrophenylhydrazine (DNPH), trichloroacetic acid (TCA), 1,1,3,3-tetraethoxypropane (TEP), 4-HNE, and 4-HHE were bought from Sigma-Aldrich (St. Louis, MO, USA). The standards of 37 fatty acid methyl esters were purchased from NU-CHEK (Minnesota, USA). The o-phenylenediamine (o-PDA) was bought from J&K Scientific Ltd (Beijing, China). Furthermore, SO (Jinlongyu, China), PO (Julong, China), OO (Borges, Spain), and LO (Jinluo, China) were purchased from local shops (Tianjin, China) and were stored at −40°C until use. Water was produced using a Milli-Q Ultrapure Water System. All other chemicals were of analytical grade.

### Thermal Treatment of Edible Oils

A dry bath incubator was used for the thermal treatment of the different oil samples (16). First, 5ml unheated SO, PO, OO, and LO were poured into an 10ml loosely closed glass bottle keeping the ratio air/sample was 1:1. And then heated to 100, 120, 150, 180, and 200°C for 30 min using the dry bath incubator (SBH200D/3, Stuart, England). Next, the heated oils were refrigerated to room temperature and stored (−40°C).

### Determination of Fatty Acid Content

The samples were methylated as studied previously (17), after which samples were analyzed using gas chromatography–mass spectrometry (GC/MS) (GC7890B-5977A, Agilent Technologies, Santa Clara, CA, USA) with some modifications (18). A capillary column (HP-5; film thickness 30 m × 0.25 μm × 0.25 μm; Agilent) was used. The injection volume was set at 1 μL and the injector temperature was set at 250°C. Helium was used as the carrier gas at a flow rate of 1.5 ml/min. GC condition program was run as follows: 120°C (maintained for 4 min) followed by warming to 175°C at a rate of 10°C/min maintained for 6 min. Then from 175°C to 210°C at 1°C/min and maintained for 5 min and from 210°C to 230°C at 10°C/min and maintained for 5 min. Alternatively, the MS program was as follows: transfer line and ion source temperatures at 200°C, scan range at m/z 30–500, and positive electron impact ionization at an ionization potential of 70 eV. Peaks generated by GC-MS were identified by the mass spectral characteristics and confirmed by comparison with a reference mixture of 37 fatty acids methyl esters. The incomplete methylation using the methanolic potassium hydroxide method causes the low fatty acid data. Hence, we chose peak area normalization as the quantitative method of fatty acid. The relative content of fatty acid in oil samples, expressed as a percentage, was determined by calculating the area of the corresponding fatty acid peak with appropriate normalization to the sum of the areas of all the fatty acid peaks.

### Determination of α-DCs

After α-DCs of oils is captured using o-PDA during thermal treatment, derivatives were determined using a high-performance liquid chromatography-mass spectrometer (HPLC-MS, Agilent 1200 LC/MSD-Trap-XCT system, Agilent Technologies, Santa Clara, CA, USA) as previously reported (19).

### Determination of MDA, 4-HHE, and 4-HNE

Analysis of MDA, 4-HNE, and 4-HNE concentrations was then conducted according to a previously described method (20). The preparation of 10 mg/ml-MDA was procured through the hydrolysis of TEP in a 5%-TCA solution. The standard working solutions of (0.02 ~ 10 mg/L for MDA, 4-HNE, and 4-HNE) were prepared using ethanol/water 1:1 (v/v). Then 2 g oil was mixed with 2.5% (w/v) TCA and DNPH [0.05 mol/L in ethanol/HCl 12 M 9:1 (v/v)]. After preparing derivatives, the separation was used with a CBM-20A HPLC system (Shimadzu, Shanghai, China) equipped with an SPD-M20A detector and an ODS HYPERSIL column (4.6 × 250 mm, 5 μm) from Thermo Scientific. The UV wavelength was set at 378 and 310 nm to detect 4-HHE, 4-HNE, and MDA derivatives. The flow rate was set at 1.0 ml/min, whereas the injection volume was set at 20 μl. Furthermore, the mobile phase was acetonitrile and water (solvent A and B). The gradient elution program was 45% solvent A maintained for 0–18 min, then from 45 to 70% solvent A in 5 min, and then 70% solvent A for 15 min.

### Determination of Volatile Compounds

GC/MS was used to analyze volatile compounds after solid-phase microextraction (SPME) following a previously published procedure (21). First, 2 ml of oil samples were positioned in a headspace vial (10 ml) with a silicone septum. And then 0.1ml of the 80 mg/L of 3-methyl-3-buten-1-ol (internal standard) was introduced into the headspace vial (10 ml). The headspace vial was taken in a 40°C-water bath under magnetic stirring (30 min) and the volatile compounds were continuously captured on an SPME fiber (50/30 μm divinyl benzene/carboxen/polydimethylsiloxane coating; Supelco, Bellefonte, PA). Then, trapped volatile compounds took place in an injector at 250°C, and the compounds promptly separated. A GC/MS (ISQ 7000, Thermo Fisher Scientific, Massachusetts State, USA) system was used for separation on an HP-5MS capillary column (film thickness of 30 m × 0.25 μm × 0.25 μm). Furthermore, GC/MS was operated using the following conditions: Helium as the carrier gas at a flow rate of 1.2 ml/min; oven temperature program maintained at 40°C for 2 min, then from 40 to 150°C at 4°C/min and maintained at 150°C for 2 min; then from 150 to 250°C at 8°C/min and maintained at 250°C for 6 min; scan range at m/z 35–500. The volatile components were separated by GC, and different components formed their respective chromatographic peaks. GC-MS and computer library (NIST standard spectrum library and chemical structure formula library) were used for analysis and identification. The quantification was relative areas calculated using the ratio of sample area to internal standard area.

### Statistical Analyses

These analyses were reported as the mean ± standard deviation of three independent extractions. Statistical calculations were performed utilizing Duncan's test (Version 24.0, IBM, SPSS Inc, Armonk, NY, USA) Statistics software package. Moreover, statistical analyses were conducted using Origin 2011 (Origin Lab Corporation, Northampton, MA, USA). Differences between pairs of means with *p* < 0.05 were considered statistically significant.

## Results

### Composition and Changes in Fatty Acid During Thermal Processing

The fatty acid composition of edible oil is a crucial element accounting for the stability of lipid oxidation. The compositions of fatty acid in different saturation type oil samples (SO, PO, OO, and LO) were therefore analyzed before heating (Supplementary Table S1). The SO possessed high levels of LA (69.28%), and it was the only oil containing linolenic acid (LNA) (7.97%) of the four edible oil samples. Moreover, OO (78.44%) had the highest proportion of oleic acid (OA). Furthermore, PO and LO had higher percentages of palmitic acid (35.25%) and stearic acid (16.99%), respectively. During thermal treatment, the oil subjected to continuous heating showed changes in the distribution of fatty acids. Results of the fatty acid obtained after thermal treatment for edible oils are shown in Supplementary Table S1. As observed, the heating process changed the fatty acid content of all samples, which mainly affected their polyunsaturated fatty acids (PUFAs) fractions. Furthermore, with the increase in heating temperature, the content of saturated fatty acids (SFAs) was increased. PUFAs in SO, PO, OO, and LO decreased significantly (*p* < 0.05) from 77.26, 22.53, 11.71, and 25.56% at 100°C to 72.72, 17.98, 8.88, and 16.39% at 200°C, respectively.

### Changes in α-DCs During Thermal Processing

**Figure 1:**
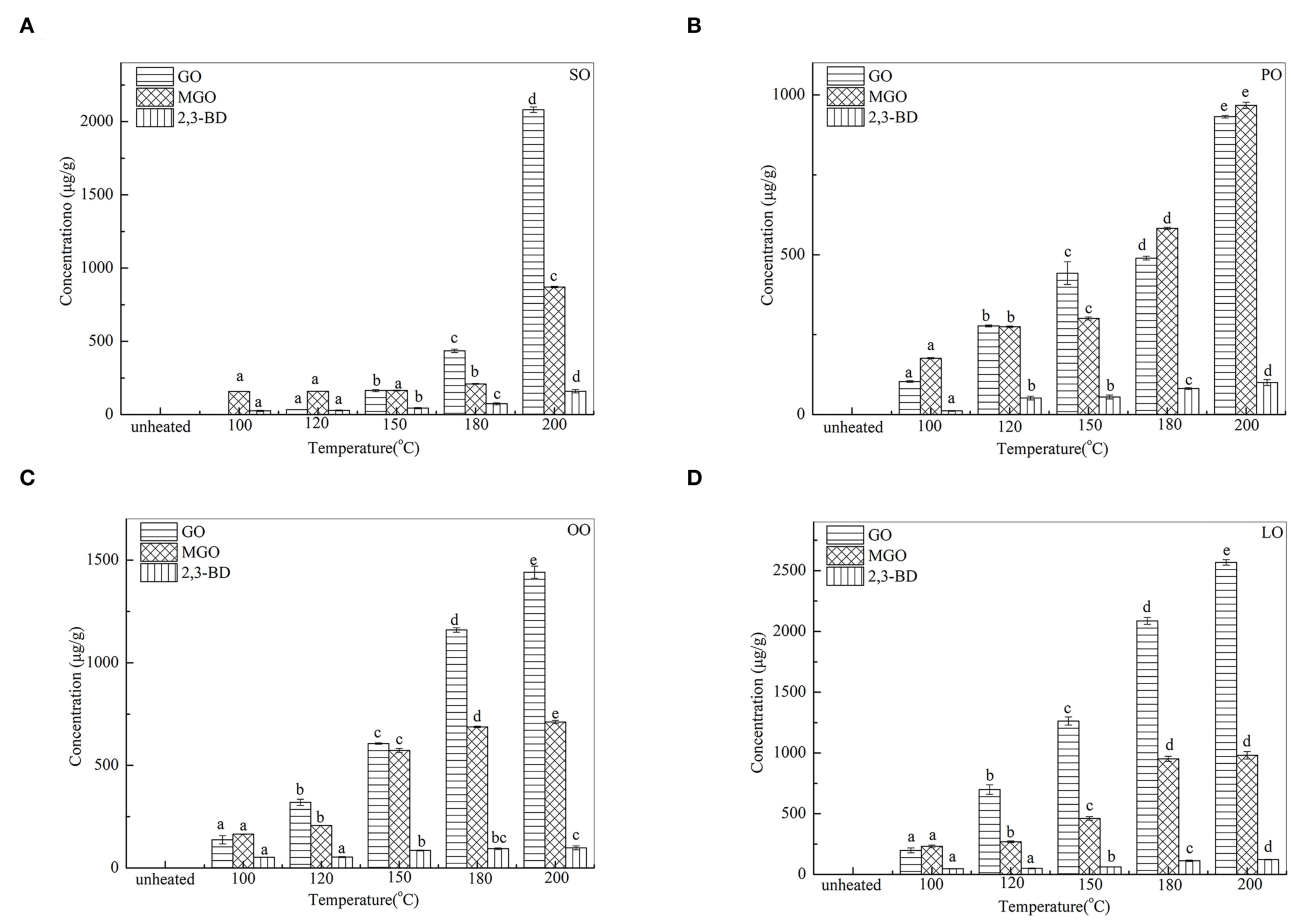
Changes of a-DCs in SO **(A)**, PO **(B)**, OO **(C)**, and LO **(D)** at different temperatures. Data were expressed as mean ± SD in triplicates. Different lowercase denotes significant difference (*p* < 0.05).

Figure 1 shows the quantities of GO, MGO, and 2,3-BD identified after heating at 100 ~ 200°C for 30 min. As observed, quantities of α-DCs identified in heated edible oil samples varied across the types and temperatures. The total quantity of α-DCs ranged from 177.4 μg/g (SO) to 476 μg/g (LO) at 100°C and from 1999.4 μg/g (PO) to 2671.2 μg/g (LO) at 200°C. Furthermore, with the temperature increased from 100°C to 200°C, total quantities of α-DCs increased 17-fold of the heated SO. However, a little increase in the temperature variation was detected in PO, OO, and LO (6.8-, 6.3-, and 7.7-fold increases, respectively). These slight variations are proposed to the different content of SFAs and PUFAs in oil samples. Results also showed that the contents of GO and MGO were the highest in OA-rich oils (LO, PO). As observed, the content of 2,3-BD was the highest in SO (LA-rich) at 200°C.

### Changes in MDA, 4-HNE, and 4-HHE During Thermal Processing

**Figure 2:**
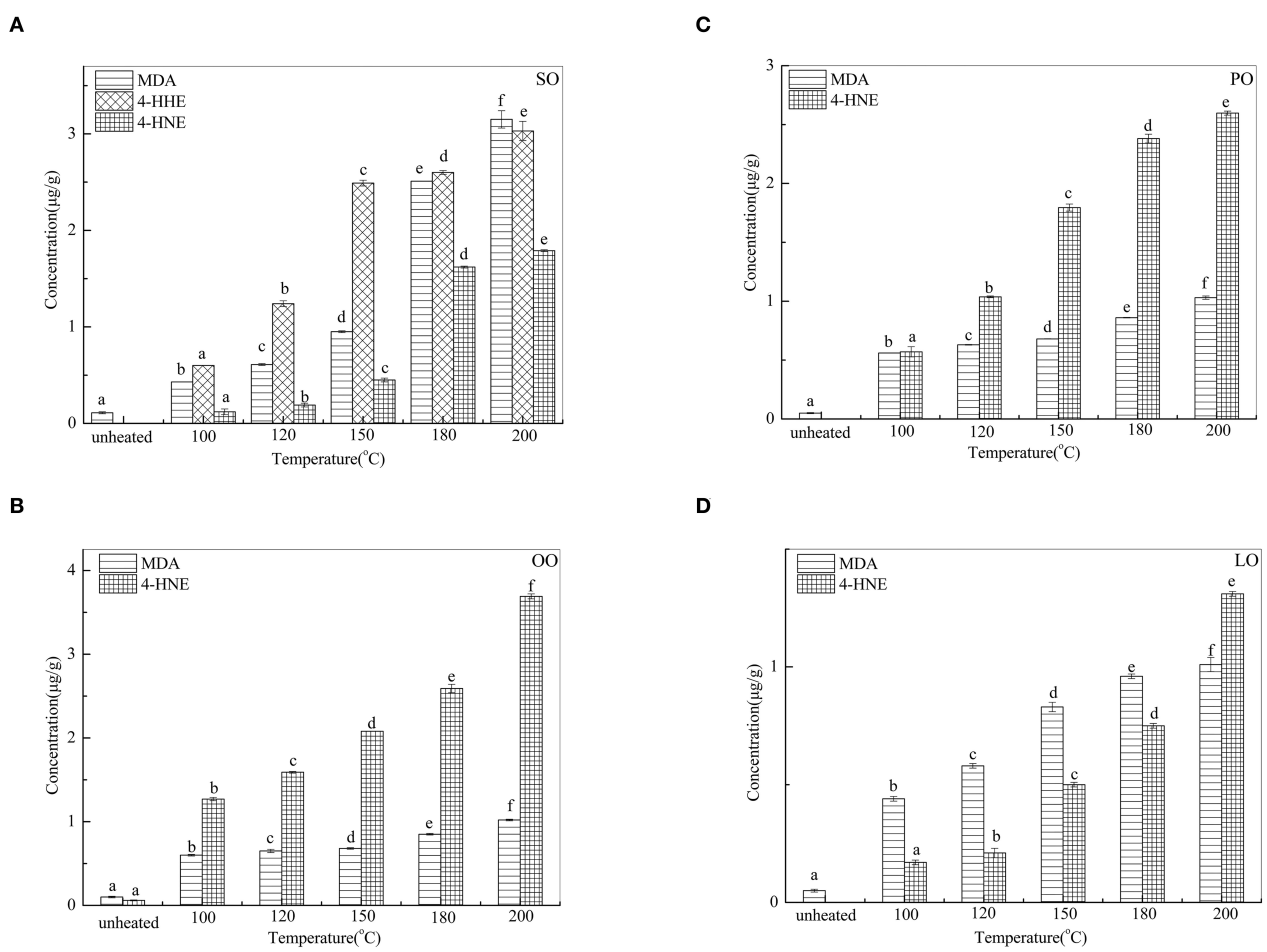
Changes of MDA, 4-HHE, and 4-HNE in SO **(A)**, PO **(B)**, OO **(C)**, and LO **(D)** at different temperatures. Data were expressed as mean ± SD in triplicates. Different lowercase denotes significant difference (*p* < 0.05).

Changes in MDA, 4-HHE, and 4-HNE are given in Figure 2. The 4-HHE was detected only in SO, and the changes of 4-HHE were increased significantly (*p* < 0.05) with the increase iN temperature. In SO, the 4-HHE concentration increased from 0.60 μg/g at 100°C to 3.03 μg/g at 200°C. In contrast, MDA and 4-HNE were detected in all oil samples heated at different temperatures. With an increase in temperature, MDA content was the highest in SO (3.15 μg/g) and 4-HNE content was the highest in OO (3.69 μg/g) at 200°C.

### Changes in Volatile Aldehyde Compounds During Thermal Processing

**Figure 3:**
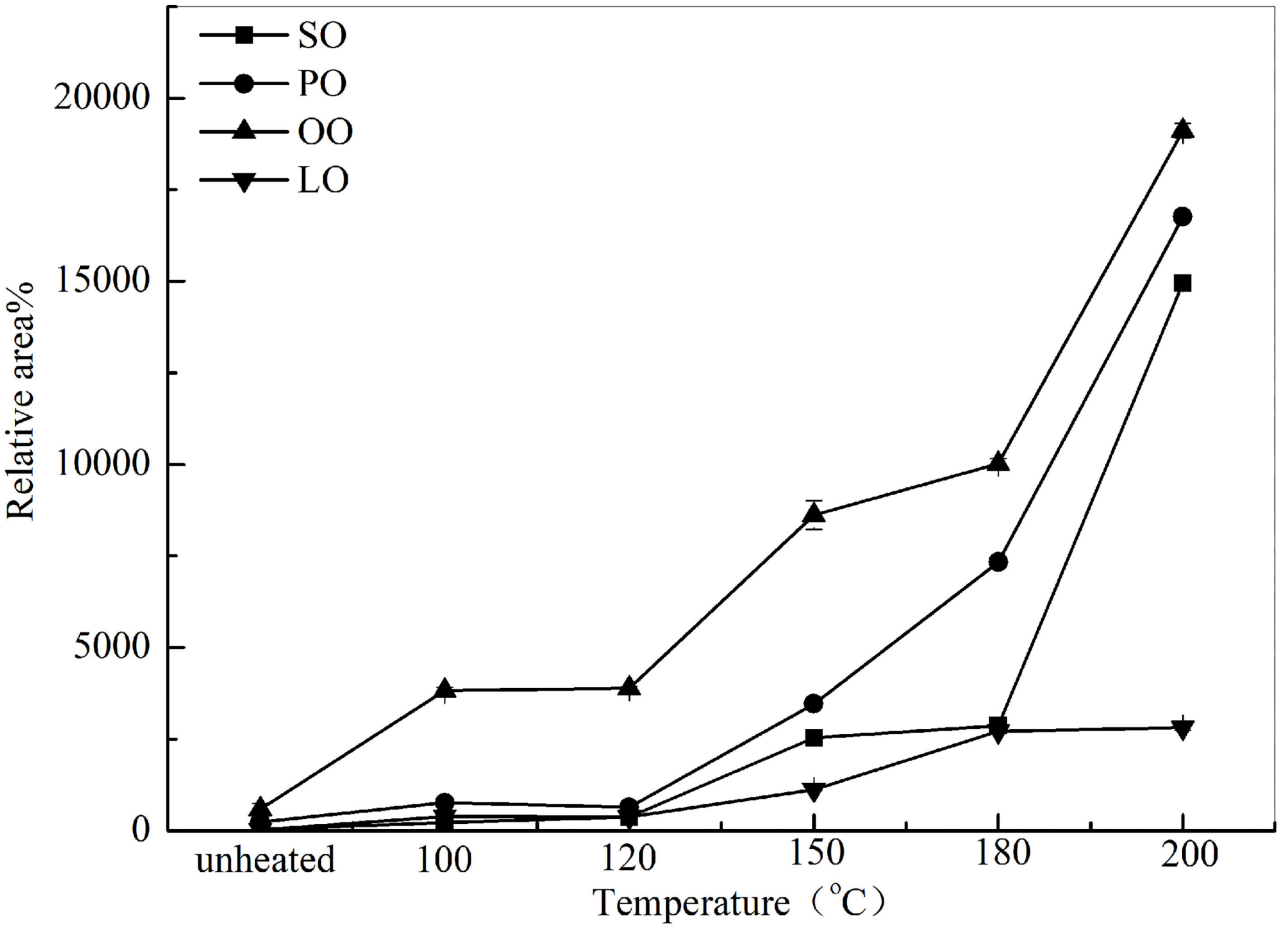
Changes of total volatile compounds in SO, PO, OO, and LO at different temperatures. Data were expressed as mean ± SD in triplicates.

The aldehydes were the major components of volatile compounds in heated oil. As shown in Figure 3, the total volatile aldehydes of SO, PO, OO, and LO increased linearly with the heating temperature, indicating that this heat processing promoted the formation of oxidation products. As observed, total volatile aldehydes in SO, PO, OO, and LO varied from 228.5, 763.61, 3828.42, 384.87% at 100°C to 14951.98, 16759.70, 19108.91, and 2818.3% at 200°C, respectively. Furthermore, although the content of total volatile aldehydes in OO was higher than that of PO, SO, and LO, the increase of total volatile aldehydes in SO was faster when heated at high temperature (180 ~ 200°C).

The 16 aldehydes were the major components of volatile compounds detected during the thermal treatment. Among the aldehydes measured, the saturated aldehydes present were butanal, hexanal, heptanal, octanal, and nonanal. The monounsaturated aldehydes were 2-butenal, 2-pentenal, 2-hexenal, 2-heptenal, 2-octenal, 2-decenal, and 2-undecenal. Then, polyunsaturated aldehydes were 2,4-heptadienal and 2, 4-decadienal. Although 2,4-decadienal (E,Z), 2,4-heptadienal (E,Z), 2,4-heptadienal (E,E), and 2-undecenal were undetected in SO heated to 100°C (Table 1), its levels increased with thermal treatment to 37.22%, 9.10%, 24.73%, and 11.38% at 120°C and to 520.99%, 1314.60%, 5250.11%, and 499.30% at 200°C. Furthermore, though 2-butenal was undetected in LO heated to 100°C, its relative content increased constantly with heating to 5.34% at 120°C and 24.35% at 200°C (Table 2). Additionally, during the thermal treatment, the 2-hexenal's content decreased significantly *(p* < 0.05) to the lowest value (515.93%) detected in OO heated at 200°C (Table 3). Results indicated that during the thermal process of the four oil samples, 2-butenal, 2-pentenal, hexanal, and 2, 4-heptadienal were distributed in SO that was rich in PUFAs and had a high content. As observed, 2-Hexenal, 2-decenal, 2, 4-decadienal, nonanal, 2-octenal, 2-heptenal, and 2-undecenal were distributed more in OA-rich OO with high concentrations. Likewise, octanal, nonanal, 2-octenal, 2-heptenal, and 2-undecenal were distributed more in PO (Table 4), which was rich in OA and palmitic acid.

**Table 1:** Changes in the volatile oxidation products of SO at different heating temperatures.

**Compounds**		**Concentration (internal standard area %)**					
		**Unheated**	**100**°**C**	**120**°**C**	**150**°**C**	**180**°**C**	**200**°**C**
SO	2-Butenal	ND	3.04 ± 0.08^a^	7.23 ± 1.71^a^	33.27 ± 1.37^b^	70.45 ± 4.34^c^	292.18 ± 15.13^d^
	Pentanal	21.41 ± 0.81^a^	34.02 ± 5.27^a^	15.51 ± 2.25^a^	27.26 ± 8.06^a^	61.60 ± 7.59^b^	214.4 ± 19.18^c^
	2-Pentenal	ND	4.16 ± 0.30^a^	7.69 ± 2.45^a^	16.42 ± 0.53^b^	29.62 ± 0.47^c^	102.26 ± 2.81^d^
	Hexanal	34.91 ± 6.70^a^	119.29 ± 2.27^c^	54.66 ± 1.33^ab^	98.74 ± 17.57^bc^	239.99 ± 27.41^d^	898.72 ± 66.76^e^
	2-Hexenal	ND	2.29 ± 0.31^a^	3.82 ± 1.09^a^	23.42 ± 1.43^b^	40.60 ± 2.94^c^	146.21 ± 8.28^d^
	Heptanal	6.88 ± 0.33^a^	11.16 ± 0.13^a^	5.16 ± 1.27^a^	15.00 ± 4.74^a^	21.81 ± 3.37^a^	108.10 ± 22.12^b^
	2-Heptenal	4.71 ± 0.21^a^	27.97 ± 1.28^ab^	92.87 ± 19.25^b^	254.93 ± 43.30^c^	325.44 ± 30.70^c^	1348.16 ± 52.19^d^
	2,4-Heptadienal, (E,Z)	ND	ND	37.22 ± 7.53^a^	127.15 ± 29.84^b^	128.72 ± 14.27^b^	520.99 ± 41.57^c^
	Octanal	1.61 ± 0.69^a^	3.57 ± 0.37^a^	5.63 ± 2.32^ab^	10.77 ± 0.96^ab^	15.26 ± 8.94^c^	101.36 ± 3.73^d^
	2,4-Heptadienal, (E,E)	ND	6.20 ± 0.49^a^	61.63 ± 14.01^a^	420.87 ± 59.33^b^	373.88 ± 9.61^b^	2182.05 ± 43.01^c^
	2-Octenal	ND	3.10 ± 0.49^a^	10.4 ± 3.84^a^	52.8 ± 17.61^b^	78.76 ± 14.05^b^	462.63 ± 1.32^c^
	Nonanal	4.97 ± 2.72^a^	12.16 ± 2.85^a^	19.85 ± 7.37^a^	43.35 ± 1.77^ab^	89.50 ± 1.15^b^	783.21 ± 51.96^c^
	2-Decenal	ND	1.54 ± 0.41^a^	13.07 ± 3.68^a^	48.72 ± 3.10^ab^	84.81 ± 23.87^b^	727.70 ± 32.34^c^
	2,4-Decadienal, (E,Z)	ND	ND	9.10 ± 2.88^a^	271.20 ± 75.03^a^	271.88 ± 8.71^a^	1314.60 ± 515.74^b^
	2,4-Decadienal, (E,E)	ND	ND	24.73 ± 5.88^a^	1041.39 ± 168.98^b^	920.04 ± 23.07^b^	5250.11 ± 568.49^c^
	2-Undecenal	ND	ND	11.38 ± 3.61^a^	46.47 ± 2.14^a^	109.23 ± 7.42^b^	499.30 ± 50.49^c^

*SO, soybean oil. Data are mean value of triplicate with SD. Different lowercase denotes a significant difference (p < 0.05) in the same row*.

**Table 2:** Changes in the volatile oxidation products of LO at different heating temperatures.

**Compounds**		**Concentration (internal standard area %)**					
		**unheated**	**100**°**C**	**120**°**C**	**150**°**C**	**180**°**C**	**200**°**C**
LO	2-Butenal	ND	ND	5.34 ± 4.14^a^	11.32 ± 1.36^a^	24.44 ± 8.73^b^	24.35 ± 3.59^b^
	Pentanal	ND	14.82 ± 0.14^a^	14.89 ± 1.93^a^	26.92 ± 4.70^a^	53.04 ± 4.43^b^	52.60 ± 19.21^b^
	2-Pentenal	ND	2.06 ± 0.27^a^	2.84 ± 1.46^a^	8.98 ± 0.01^b^	14.42 ± 1.68^bc^	15.90 ± 4.27^c^
	Hexanal	10.78 ± 2.60^a^	73.47 ± 3.9^b^	75.29 ± 6.12^b^	108.98 ± 21.75^bc^	187.55 ± 11.16^d^	149.72 ± 46.65^cd^
	2-Hexenal	ND	4.12 ± 0.06^a^	4.60 ± 0.59^a^	23.87 ± 3.71^b^	33.62 ± 5.85^b^	38.65 ± 13.33^b^
	Heptanal	ND	12.72 ± 0.21^a^	11.53 ± 1.53^a^	18.19 ± 2.48^a^	37.78 ± 2.22^b^	35.56 ± 10.94^b^
	2-Heptenal	6.32± 0.58^a^	62.15 ± 4.5^b^	67.64 ± 7.75^b^	183.02 ± 28.64^c^	237.05 ± 43.48^c^	279.13 ± 82.29^c^
	2,4-Heptadienal, (E,Z)	ND	5.12 ± 1.22^a^	4.38 ± 1.14^a^	29.2 ± 3.73^ab^	49.8 ± 12.12^bc^	64.89 ± 16.65^c^
	Octanal	ND	19.10 ± 0.96^a^	15.32 ± 1.04^ab^	19.38 ± 4.13^ab^	39.97 ± 0.20^b^	31.44 ± 10.66^c^
	2,4-Heptadienal, (E,E)	ND	8.99 ± 0.86^a^	9.65 ± 1.65^a^	91.60 ± 9.14^b^	173.15 ± 34.34^c^	219.74 ± 34.02^c^
	2-Octenal	ND	25.11 ± 2.5^a^	22.11 ± 6.06^a^	68.89 ± 13.22^b^	96.46 ± 18.17^b^	105.25 ± 28.06^b^
	Nonanal	ND	40.23 ± 1.9^a^	36.84 ± 5.05^a^	86.00 ± 20.44^a^	173.5 ± 21.35^b^	160.62 ± 48.18^b^
	2-Decenal	ND	35.01 ± 3.08^a^	29.22 ± 8.28^a^	71.13 ± 7.68^b^	216.46 ± 7.05^c^	230.98 ± 28.04^c^
	2,4-Decadienal, (E,Z)	ND	10.83 ± 1.08^a^	9.89 ± 4.78^a^	72.54 ± 3.39^b^	251.06 ± 2.88^c^	257.21 ± 3.88^c^
	2,4-Decadienal, (E,E)	ND	44.64 ± 1.01^a^	36.86 ± 15.43^a^	238.21 ± 17.47^b^	923.49 ± 31.19^c^	955.73 ± 10.58^c^
	2-Undecenal	ND	26.63 ± 5.33^a^	24.00 ± 6.83^a^	58.83 ± 0.59^b^	202.84 ± 20.12^c^	196.57 ± 4.99^c^

*LO, lard oil. Data are mean value of triplicate with standard deviation. Different lowercase denotes significant difference (p < 0.05) in the same row*.

**Table 3:** Changes in the volatile oxidation products of OO at different heating temperatures.

**Compounds**		**Concentration (internal standard area %)**					
		**Unheated**	**100**°**C**	**120**°**C**	**150**°**C**	**180**°**C**	**200**°**C**
OO	2-Butenal	3.16 ± 0.41^a^	21.41 ± 1.12^b^	34.95 ± 1.03^bc^	46.46 ± 11.55^c^	82.65 ± 2.43^d^	101.78 ± 10.73^e^
	Pentanal	ND	49.87 ± 8.55^a^	72.97 ± 4.32^ab^	91.2 ± 3.67^b^	139.07 ± 7.35^c^	224.31 ± 24.92^d^
	2-Pentenal	ND	26.78 ± 3.03^a^	36.08 ± 0.78^ab^	43.70 ± 13.50^ab^	44.01 ± 2.01^ab^	53.2 ± 6.03^b^
	Hexanal	46.64 ± 13.59^a^	293.46 ± 82.19^b^	309.36 ± 5.94^b^	414.13 ± 95.49^bc^	490.35 ± 28.21^c^	591.25 ± 150.56^c^
	2-Hexenal	512 ± 134.38^a^	2405.24 ± 98.4^d^	1627.4 ± 94.73^c^	1339.98 ± 215.2^bc^	934.18 ± 23.36^ab^	515.93 ± 16.27^a^
	Heptanal	5.14 ± 0.98^a^	63.98 ± 4.57^ab^	80.35 ± 30^ab^	146.52 ± 53.01^bc^	218.75 ± 72.57^cd^	296.9 ± 29.49^d^
	2-Heptenal	16.66 ± 2.82^a^	228.13 ± 2.55^b^	458.5 ± 36.7^c^	630.39 ± 114.37^d^	759.17 ± 9.41^e^	873.28 ± 9.22^e^
	2,4-Heptadienal, (E,Z)	ND	ND	77.91 ± 6.81^a^	427.98 ± 31.26^b^	422.81 ± 10.15^b^	473.44 ± 32.01^b^
	Octanal	6.26 ± 1.22^a^	41.48 ± 1.19^ab^	64.26 ± 0.1^b^	86.87 ± 21.54^b^	179.18 ± 18.6^c^	337.89 ± 83.4^d^
	2,4-Heptadienal, (E,E)	ND	32.00 ± 0.06^a^	53.43 ± 8.12^a^	252.2 ± 39.29^b^	322.26 ± 40.47^c^	625.06 ± 17.06^d^
	2-Octenal	ND	24.29 ± 3.33^a^	56.9 ± 1.24^a^	210.75 ± 54.29^b^	303.8 ± 40.33^c^	435.7 ± 9.99^d^
	Nonanal	ND	528.35 ± 31.4^a^	796.25 ± 8.41^b^	1292.11 ± 41.34^c^	1501.72 ± 117.2^d^	2129.91 ± 70.88^e^
	2-Decenal	5.86 ± 1.30^a^	45.00 ± 1.01^ab^	80.24 ± 0.16^ab^	388.74 ± 103.04^b^	978.08 ± 246.14^c^	2622.2 ± 143.91^d^
	2,4-Decadienal, (E,Z)	ND	27.26 ± 0.59^a^	42.93 ± 2.22^a^	1091.58 ± 93.77^b^	1127.93 ± 61.06^b^	2642.72 ± 149.4^c^
	2,4-Decadienal, (E,E)	ND	41.17 ± 1.52^a^	74.6 ± 6.93^a^	2072.75 ± 569.9^b^	2227.81 ± 71.72^b^	5800.48 ± 44.2^c^
	2-Undecenal	ND	ND	22.98 ± 3.53^a^	85.44 ± 18.85^a^	290.97 ± 66.04^b^	1384.86 ± 98.71^c^

*OO, olive oil. Data are mean value of triplicate with SD. Different lowercase denotes significant difference (p < 0.05) in the same row*.

**Table 4:** Changes in the volatile oxidation products of PO at different heating temperatures.

**Compounds**		**Concentration (internal standard area %)**					
		**Unheated**	**100**°**C**	**120**°**C**	**150**°**C**	**180**°**C**	**200**°**C**
PO	2-Butenal	ND	3.46 ± 2.14^a^	3.76 ± 1.03^a^	8.00 ± 0.15^a^	27.41 ± 1.22^b^	79.73 ± 13.11^c^
	Pentanal	ND	3.03 ± 1.18^a^	12.44 ± 3.13^a^	33.87 ± 0.78^a^	154.62 ± 22.22^b^	256.11 ± 45.18^c^
	2-Pentenal	ND	2.91 ± 0.18^a^	3.01 ± 0.03^a^	8.62 ± 0.04^a^	41.41 ± 4.24^b^	47.14 ± 5.96^b^
	Hexanal	162.99 ± 13.87^a^	345.59 ± 5.95^b^	171.99 ± 37.68^a^	182.55 ± 13.22^a^	695.76 ± 0.16^c^	732.63 ± 93.20^c^
	2-Hexenal	ND	5.06 ± 0.42^a^	8.98 ± 3.50^a^b	14.83 ± 1.42^b^	71.12 ± 2.53^c^	98.36 ± 6.43^d^
	Heptanal	22.10 ± 1.05^a^	55.62 ± 1.16^a^	22.45 ± 2.96^a^	21.73 ± 1.90^a^	129.80 ± 21.31^b^	287.84 ± 47.40^c^
	2-Heptenal	11.99 ± 0.17^a^	107.71 ± 7.27^b^	102.69 ± 21.65^b^	126.34 ± 5.33^b^	756.86 ± 95.90^c^	801.38 ± 21.64^c^
	2,4-Heptadienal, (E,Z)	ND	27.57 ± 1.43^a^	30.22 ± 11.14^a^	103.84 ± 13.29^b^	253.16 ± 51.76^c^	215.00 ± 21.72^c^
	Octanal	18.17 ± 1.26^a^	53.96 ± 4.51^ab^	26.10 ± 8.72^a^	25.53 ± 2.55^a^	115.00 ± 12.51^b^	477.36 ± 66.91^c^
	2,4-Heptadienal, (E,E)	2.43 ± 0.61^a^	15.58 ± 3.01^a^	19.46 ± 2.26^a^	115.41 ± 8.60^b^	223.90 ± 43.15^c^	487.31 ± 19.37^d^
	2-Octenal	ND	32.30 ± 0.52^a^	39.14 ± 8.00^a^	62.80 ± 4.65^a^	200.34 ± 34.27^b^	401.07 ± 9.19^c^
	Nonanal	18.55 ± 1.28^a^	81.21 ± 4.04^a^	83.13 ± 27.83^a^	217.97 ± 10.45^a^	1495.71 ± 153.94^b^	2315.11 ± 180.01^c^
	2-Decenal	ND	13.34 ± 0.89^a^	18.46 ± 1.32^a^	101.7 ± 14.16^a^	118.31 ± 8.61^a^	1992.83 ± 474.26^b^
	2,4-Decadienal, (E,Z)	ND	4.5 ± 0.56^a^	31.90 ± 5.03^a^	783.2 ± 114.53^b^	903.66 ± 78.64^b^	2160.22 ± 351.97^c^
	2,4-Decadienal, (E,E)	3.16 ± 1.42^a^	8.84 ± 1.91^a^	47.15 ± 0.01^a^	1611.37 ± 232.95^b^	2034.05 ± 121.03^b^	5014.11 ± 868.53^c^
	2-Undecenal	ND	2.95 ± 0.08^a^	15.46 ± 0.42^a^	43.7 ± 7.75^a^	107.99 ± 4.76^a^	1393.54 ± 473.91^b^

*PO, palm oil. Data are mean value of triplicate with SD. Different lowercase denotes significant difference (p < 0.05) in the same row*.

### The Relationship Between LOPs and Fatty Acid

In this study, Pearson's correlation coefficient analysis was used to further study the correlation between LOPs and fatty acids of oil during heating. Supplementary Tables S2–S5 shows the statistical significance levels (showed as the asterisk ^*^) and Supplementary Figure S1 shows the hierarchical clustering heat map of LOPs and fatty acids. Results showed that the LOPs had a highly negative correlation with PUFAs, indicating that the formation of LOPs can be attributed to the decrease in PUFAs. Furthermore, a significant correlation between myristic acid, 2, 4-heptadienal, and GO was observed (*p* < 0.01). LNA was significantly correlated with MDA and 4-HHE (*p* < 0.01), SFAs and PUFAs were significantly correlated with 4-HHE and MDA, respectively (*p* < 0.01).

## Discussion

The effect of heating temperature and fatty acid types on the formation of LOPs was investigated by heating the different saturation oil at different temperatures. The heating temperature (100, 120, 150, 180, and 200°C) simulate the temperature of different types of cooking methods (steaming, boiling, stir-frying, frying and roasting) to explore the effect of cooking temperature on LOPs. Given that the LOPs were increased with the heating temperature, the oil with different saturation heated at the 200°C (frying and roasting temperature) was chosen for comparison with the oil heated at the 100°C (steaming, boiling). The contents of MGO, 2,3-BD, MDA, 4-HNE, 4-HHE total volatile aldehydes were decreased by 81.86, 83.87, 86.35, 93.29, 80.20, and 98.47% in SO, respectively. The contents of GO, MGO, 2,3-BD, MDA, 4-HNE, and total volatile aldehydes were decreased by 88.95, 81.80, 88.37, 45.63, 78.00, and 95.45% in PO, respectively. The contents of GO, MGO, 2,3-BD, MDA, 4-HNE, and total volatile aldehydes were decreased by 90.48, 76.80, 45.92, 41.18, 65.58, and 79.97% in OO, respectively. The contents of GO, MGO, 2,3-BD, MDA, 4-HNE, and total volatile aldehydes were decreased by 92.33, 76.40, 61.47, 56.44, 87.02, and 53.05% in LO, respectively. Hence, we should appropriately reduce the heating temperature of oils to avoid increasing the amounts of harmful compounds being generated at high temperatures.

Our analysis of SO, PO, OO, and LO at different heating temperatures showed that the concentration of fatty acid changed with temperature. A similar finding was observed in a previous study (22). The finding shows that SFAs were not only used as a product to increase the content but also as a reaction substrate to participate in the whole frying process. Notably, a decrease in PUFAs content was observed with the increased temperature in oil samples. PUFAs oxidation was the major account for the improvement of SFAs in edible oils during thermal processing. During thermal treatment, the decrease in PUFAs was due to their instability during thermal processing (23). As observed, PUFAs through fission and oxidation generated SFAs and monounsaturated fatty acids (MUFAs) coincided with oxidative product formation, and SFAs had better stability (24). The higher the fatty acid unsaturation, the more oxidative degradation occurs during thermal processing (14).

The results showed that the composition of fatty acids in oil affected greatly the generation of LOPs during heat treatment. The generation of LOPs was correlated with the attacked positions of hydroperoxides of UFAs that can produce several hydroperoxide isomers. The fracture of the OA chain, resulting in the loss of an H from C, followed by the absorption of the hydrogen peroxide free group (OOH) and produced 8-hydroperoxides, 9-hydroperoxides, 10-hydroperoxides, and 11-hydroperoxides (25, 26). LA could produce 9-hydroperoxides, 10-hydroperoxides, and 13-hydroperoxides (27) and LNA generated 9-hydroperoxides, 12-hydroperoxides, 13-hydroperoxides, and 16-hydroperoxides during oxidation of oil (11, 25). 4-HNE, 4-HHE, 2-pentanal, and hexanal most likely originate from the breakdown of the 9-hydroperoxides, 12-hydroperoxides, 13-hydroperoxides, and 12/13-hydroperoxides separately (11, 26). This explains the high level of 4-HNE in OO (rich in MUFAs) and more content of 4-HHE, 2-pentanal, and hexanal in SO (rich in PUFAs). In addition, LNA can form MDA through β-scission and molecular rearrangement, thus MDA is generated more in SO (rich in LNA) (28).

The contents and types of LOPs are closely related to the composition of fatty acids in edible oil. In the light of these results, it can be concluded that the higher the UFAs content, the higher the content of LOPs produced during the heating process. Interestingly, the LOPs found in SO at 100°C were much < those in the other oils at 100°C, whereas it formed faster than those oils at 200°C. The energy required for the degradation of fatty acids was directly proportional to its saturation due to more reaction procedures required for fatty acids with unsaturated C–C bonds to breakdown into lower molecular weight compounds (12). In this study, high associations between aldehyde concentrations and fatty acids were observed in the four edible oil samples (Supplementary Figure S1). Different types of fatty acids had varying relationships to different types of LOPs. For instance, hexanal was a typical volatile oxidation product of LA, and the content of octanal, nonanal, and undecanal was high in OA-rich oils (3, 29). The close relationship between the oxidation products generated and the degradation of UFAs. For example, heptanal, octanal, nonanal, and 2-decenal have a close correlation with OA (10); pentanal, hexanal, 2-octenal, 2-decenal and 2,4-decadienal have a close correlation with LA; 2-butenal 2-pentenal, 2-hexenal, 2-heptenal, and 2,4-heptadienal have a close correlation with LNA (15, 30). On the basis of the relationships between fatty acid types and LOPs, it may be available to speculate on the quantity of different LOPs during thermal processing. In addition, to avoid the amounts of harmful lipid oxidation compounds being generated more during thermal treatment, choose different kinds of edible oil for different cooking methods. When the cooking methods were the steaming and boiling, choosing the oil rich in UFA can reduce the generation of LOPs, and for the frying and roasting conditions, choosing the oil rich in SFA can reduce the generation of LOPs.

## Conclusion

Results of this study demonstrated that edible oils produced adverse LOPs during high-temperature treatments, and the LOPs increased significantly (*p* < 0.05) when the temperature increased from 100 to 200°C. As observed, different fatty acids also had varying effects on different LOPs. The results showed that LNA was the source fatty acid of 4-HHE. As shown, 2-butenal, 2-pentenal, hexanal, 2,4-heptadienal, 2,3-BD, MDA, and 4-HHE were distributed more in SO rich in PUFAs. Results also showed that 2-hexenal, 2-decenal, 2,4-decadienal, and 4-HNE were distributed in OA-rich oils (OO) having high concentrations. Besides, both octanal and MGO were distributed more in highly saturated PO (rich in OA and palmitic acid). GO was distributed more in LO (rich in OA and LA). In summary, the high temperature and the loss of UFAs could lead to an increase in hazardous LOPs of heating edible oils, thereby increasing the potential risk to human health. Therefore, we should appropriately reduce the heating temperature of oils to avoid increasing the amounts of harmful compounds being generated at high temperatures.

## Data Availability Statement

The original contributions presented in the study are included in the article/Supplementary Material, further inquiries can be directed to the corresponding author.

## Author Contributions

YZhu: writing-original draft and investigation. YZha, JD, and XH: data curation. JW, CL, LD, and YZ: conceptualization, methodology, validation, and editing. LD, HW, YY, and SW: writing-reviewing. SW: supervision. All authors contributed to the article and approved the submitted version.

## Funding

This work was supported by grants from the National Natural Science Foundation of China (No. 32030083).

## Conflict of Interest

The authors declare that the research was conducted in the absence of any commercial or financial relationships that could be construed as a potential conflict of interest.

## Publisher's Note

All claims expressed in this article are solely those of the authors and do not necessarily represent those of their affiliated organizations, or those of the publisher, the editors and the reviewers. Any product that may be evaluated in this article, or claim that may be made by its manufacturer, is not guaranteed or endorsed by the publisher.
