# Supplementary material for: Impact of Heating Temperature and Fatty Acid Type on the Formation of Lipid Oxidation Products During Thermal Processing
Source: Front Nutr. 2022 Jun 2;9:913297. doi: 10.3389/fnut.2022.913297 (PMC9201814; doi:10.3389/fnut.2022.913297)
Supplement: Supplementary file 1 [file Data_Sheet_1.docx]

Supplementary Material

# Supplementary Tables

**Supplement Table 1.** Fatty acid changes of SO, PO, OO and LO at different heating temperatures.

| Fatty acid | | Concentration(%) | | | | | |
| --- | --- | --- | --- | --- | --- | --- | --- |
|  |  | unheated | 100^o^C | 120^o^C | 150^o^C | 180^o^C | 200^o^C |
| SO | C16:0 | 4.73±0.05^a^ | 4.74±0.01^a^ | 5.10±0.06^b^ | 5.42±0.11^c^ | 5.80±0.16^d^ | 6.06±0.08^e^ |
|  | C18:2 | 69.28±0.01^c^ | 69.27±0.64^c^ | 68.39±0.16^bc^ | 67.35±0.52^b^ | 66.23±0.57^a^ | 65.50±0.27^a^ |
|  | C18:3 | 7.97±0.06^c^ | 7.97±0.20^c^ | 7.87±0.25^bc^ | 7.84±0.09^bc^ | 7.53±0.01^ab^ | 7.22±0.04^a^ |
|  | C18:1 | 14.53±0.01^a^ | 14.50±0.35^a^ | 14.85±0.08^ab^ | 15.27±0.13^b^ | 15.97±0.32^c^ | 16.43±0.21^c^ |
|  | C18:0 | 3.49±0.01^a^ | 3.53±0.10^ab^ | 3.80±0.11^b^ | 4.12±0.20^c^ | 4.48±0.09^d^ | 4.79±0.07^e^ |
|  | SFA | 8.22±0.06^a^ | 8.27±0.08^a^ | 8.90±0.16^b^ | 9.54±0.31^c^ | 10.28±0.25^d^ | 10.86±0.02^e^ |
|  | MUFA | 14.53±0.01^a^ | 14.50±0.35^a^ | 14.85±0.08^ab^ | 15.27±0.13^b^ | 15.97±0.32^c^ | 16.43±0.21^c^ |
|  | PUFA | 77.26±0.05^e^ | 77.23±0.44^e^ | 76.26±0.09^d^ | 75.19±0.44^c^ | 73.76±0.57^b^ | 72.72±0.23^a^ |
| PO | C14:0 | 0.79±0.01^a^ | 0.78±0.01^a^ | 0.83±0.02^ab^ | 0.86±0.01^b^ | 0.92±0.04^c^ | 1.02±0.01^d^ |
|  | C16:0 | 35.25±0.07^a^ | 35.24±0.11^a^ | 35.59±0.04^b^ | 36.15±0.18^c^ | 36.72±0.18^d^ | 36.18±0.03^e^ |
|  | C18:2 | 17.53±0.01^e^ | 17.51±0.08^e^ | 16.50±0.21^d^ | 15.20±0.07^c^ | 13.88±0.04^b^ | 12.98±0.31^a^ |
|  | C18:1 | 42.29±0.05^a^ | 42.29±0.07^a^ | 42.76±0.25^b^ | 43.33±0.08^c^ | 43.88±0.11^d^ | 44.06±0.34^d^ |
|  | C18:0 | 4.16±0.01^a^ | 4.19±0.11^ab^ | 4.34±0.05^bc^ | 4.47±0.03^cd^ | 4.61±0.17^d^ | 4.77±0.04^e^ |
|  | SFA | 30.19±0.06^a^ | 30.21±0.01^a^ | 30.75±0.06^b^ | 31.47±0.16^c^ | 32.24±0.06^d^ | 32.96±0.03^e^ |
|  | MUFA | 47.29±0.05^a^ | 47.29±0.07^a^ | 47.76±0.25^b^ | 48.33±0.08^c^ | 48.88±0.11^d^ | 49.06±0.34^d^ |
|  | PUFA | 22.53±0.01^e^ | 22.51±0.08^e^ | 21.50±0.21^d^ | 20.20±0.07^c^ | 18.88±0.04^b^ | 17.98±0.31^a^ |
| OO | C16:1 | 0.72±0.04^a^ | 0.72±0.03^a^ | 0.80±0.02^ab^ | 0.85±0.02^bc^ | 0.89±0.02^c^ | 0.90±0.05^c^ |
|  | C16:0 | 6.40±0.01^a^ | 6.40±0.01^a^ | 7.17±0.19^b^ | 7.83±0.18^c^ | 7.89±0.08^c^ | 8.35±0.29^d^ |
|  | C18:2 | 11.71±0.13^c^ | 11.70±0.18^c^ | 11.20±0.38^c^ | 10.41±1.29^b^ | 9.89±0.24^b^ | 8.88±0.19^a^ |
|  | C18:1 | 78.44±0.23^bc^ | 78.45±0.12^bc^ | 78.03±0.12^a^ | 78.06±0.10^ab^ | 78.35±0.16^ab^ | 78.78±0.13^c^ |
|  | C18:0 | 2.74±0.07^a^ | 2.74±0.07^a^ | 2.81±0.06^a^ | 2.87±0.06^ab^ | 3.00±0.01^bc^ | 3.11±0.02^c^ |
|  | SFA | 9.14±0.06^a^ | 9.14±0.09^a^ | 9.98±0.24^b^ | 10.69±0.23^c^ | 10.88±0.10^c^ | 11.46±0.28^d^ |
|  | MUFA | 79.16±0.18^b^ | 79.17±0.09^b^ | 78.82±0.14^a^ | 78.91±0.13^ab^ | 79.22±0.14^b^ | 79.67±0.08^c^ |
|  | PUFA | 11.71±0.13^c^ | 11.70±0.18^c^ | 11.20±0.38^c^ | 10.41±0.36^b^ | 9.89±0.24^b^ | 8.88±0.19^a^ |
| LD | C14:0 | 0.75±0.08^a^ | 0.74±0.16^a^ | 0.73±0.08^a^ | 1.01±0.05^ab^ | 1.21±0.11^bc^ | 1.51±0.25^c^ |
|  | C16:1 | 1.65±0.03 | 1.63±0.02 | 1.62±0.01 | 1.64±0.01 | 1.62±0.03 | 1.60±0.02 |
|  | C16:0 | 11.96±0.30^a^ | 12.07±0.22^a^ | 12.80±0.09^b^ | 13.38±0.17^b^ | 14.36±0.12^c^ | 15.48±0.39^d^ |
|  | C18:2 | 25.56±0.39^e^ | 25.57±0.36^e^ | 24.06±0.96^d^ | 21.86±0.59^c^ | 18.83±0.46^b^ | 16.39±0.07^a^ |
|  | C18:1 | 42.65±1.03 | 43.16±0.36 | 43.64±0.50 | 42.80±0.49 | 43.28±0.41 | 43.31±0.08 |
|  | C18:0 | 16.99±0.97^a^ | 16.83±0.80^a^ | 17.15±1.62^a^ | 19.31±0.01^b^ | 20.71±0.07^bc^ | 21.71±0.31^c^ |
|  | SFA | 29.69±0.76^a^ | 29.64±0.74^a^ | 30.68±1.44^a^ | 33.69±0.11^b^ | 36.27±0.08^c^ | 38.70±0.17^d^ |
|  | MUFA | 44.30±1.00 | 44.79±0.38 | 45.26±0.48 | 44.44±0.48 | 44.90±0.38 | 44.91±0.10 |
|  | PUFA | 25.56±0.39^e^ | 25.57±0.36^e^ | 24.06±0.96^d^ | 21.86±0.59^c^ | 18.83±0.46^b^ | 16.39±0.07^a^ |

Data are mean value of triplicate with SD. Different lowercase denotes significant difference (P < 0.05) in the same row.

**Supplement Table 2.** Pearson correlation coefficients between the levels of oxidation products and fatty acids during heating in SO.

|  |  | C16:0 | C18:2 | C18:3 | C18:1 | C18:0 | SFA | MUFA | PUFA |
| --- | --- | --- | --- | --- | --- | --- | --- | --- | --- |
| SO | 2-Butenal | 0.811 | -0.794 | -0.882* | 0.925* | 0.855 | 0.834 | 0.925* | -0.807 |
|  | Pentanal | 0.748 | -0.740 | -0.845 | 0.890* | 0.801 | 0.776 | 0.890* | -0.755 |
|  | 2-Pentenal | 0.826 | -0.808 | -0.893* | 0.934* | 0.867 | 0.848 | 0.934* | -0.821 |
|  | Hexanal | 0.751 | -0.741 | -0.846 | 0.891* | 0.803 | 0.778 | 0.891* | -0.756 |
|  | 2-Hexenal | 0.834 | -0.817 | -0.897* | 0.940* | 0.876 | 0.856 | 0.940* | -0.829 |
|  | Heptanal | 0.746 | -0.725 | -0.827 | 0.881* | 0.796 | 0.772 | 0.881* | -0.740 |
|  | 2-Heptenal | 0.820 | -0.794 | -0.875 | 0.924* | 0.860 | 0.841 | 0.924* | -0.806 |
|  | 2,4-Heptadienal, (E,Z) | 0.841 | -0.811 | -0.880* | 0.931* | 0.877 | 0.860 | 0.931* | -0.821 |
|  | Octanal | 0.751 | -0.724 | -0.824 | 0.878 | 0.797 | 0.775 | 0.878 | -0.738 |
|  | 2,4-Heptadienal, (E,E) | 0.797 | -0.765 | -0.846 | 0.904* | 0.838 | 0.819 | 0.904* | -0.777 |
|  | 2-Octenal | 0.781 | -0.756 | -0.849 | 0.900* | 0.825 | 0.804 | 0.900* | -0.769 |
|  | Nonanal | 0.736 | -0.709 | -0.815 | 0.868 | 0.783 | 0.760 | 0.868 | -0.724 |
|  | 2-Decenal | 0.748 | -0.721 | -0.822 | 0.876 | 0.794 | 0.772 | 0.876 | -0.735 |
|  | 2,4-Decadienal, (E,Z) | 0.819 | -0.791 | -0.866 | 0.920* | 0.859 | 0.840 | 0.920* | -0.802 |
|  | 2,4-Decadienal, (E,E) | 0.800 | -0.771 | -0.852 | 0.908* | 0.842 | 0.822 | 0.908* | -0.783 |
|  | 2-Undecenal | 0.802 | -0.783 | -0.875 | 0.918* | 0.846 | 0.825 | 0.918* | -0.797 |
|  | GO | 0.795 | -0.777 | -0.870 | 0.914* | 0.839 | 0.818 | 0.914* | -0.790 |
|  | MGO | 0.715 | -0.689 | -0.799 | 0.854 | 0.764 | 0.740 | 0.854 | -0.704 |
|  | 2,3-BD | 0.867 | -0.860 | -0.934* | 0.964** | 0.906* | 0.888* | 0.964** | -0.872 |
|  | MDA | 0.929* | -0.958* | -0.989** | 0.972** | 0.952* | 0.941* | 0.972** | -0.964** |
|  | 4-HHE | 0.970** | -0.953* | -0.963** | 0.900* | 0.958* | 0.965** | 0.900* | -0.948* |
|  | 4-HNE | 0.915* | -0.955* | -0.914* | 0.946* | 0.935* | 0.926* | 0.946* | -0.960** |

* Significance at P < 0.05. ** Significance at P < 0.01.

**Supplement Table 3.** Pearson correlation coefficients between the levels of oxidation products and fatty acids during heating in PO.

|  |  | C14:0 | C16:0 | C18:2 | C18:1 | C18:0 | SFA | MUFA | PUFA |
| --- | --- | --- | --- | --- | --- | --- | --- | --- | --- |
| PO | 2-Butenal | 0.949* | 0.849 | -0.844 | 0.595 | 0.859 | 0.865 | 0.595 | -0.844 |
|  | Pentanal | 0.973** | 0.938* | -0.928* | 0.765 | 0.930* | 0.944* | 0.765 | -0.928* |
|  | 2-Pentenal | 0.922* | 0.946* | -0.928* | 0.847 | 0.918* | 0.941* | 0.847 | -.928* |
|  | Hexanal | 0.789 | 0.803 | -0.766 | 0.678 | 0.751 | 0.793 | 0.678 | -0.766 |
|  | 2-Hexenal | 0.955* | 0.942* | -0.929* | 0.798 | 0.927* | 0.944* | 0.798 | -0.929* |
|  | Heptanal | 0.906* | 0.813 | -0.797 | 0.558 | 0.805 | 0.824 | 0.558 | -0.797 |
|  | 2-Heptenal | 0.888* | 0.914* | -0.920* | 0.821 | 0.881* | 0.908* | 0.821 | -.892* |
|  | 2,4-Heptadienal, (E,Z) | 0.846 | 0.945* | -0.926* | 0.948* | 0.905* | 0.930* | 0.948* | -0.926* |
|  | Octanal | 0.889* | 0.756 | -0.751 | 0.461 | 0.769 | 0.775 | 0.461 | -0.750 |
|  | 2,4-Heptadienal, (E,E) | 0.981** | 0.924* | -0.919* | 0.717 | 0.926* | 0.934* | 0.717 | -0.919* |
|  | 2-Octenal | 0.969** | 0.901* | -0.893* | 0.685 | 0.901* | 0.912* | 0.685 | -0.893* |
|  | Nonanal | 0.956* | 0.927* | -0.913* | 0.759 | 0.913* | 0.931* | 0.759 | -0.913* |
|  | 2-Decenal | 0.867 | 0.714 | -0.717 | 0.405 | 0.742 | 0.738 | 0.405 | -0.717 |
|  | 2,4-Decadienal, (E,Z) | 0.971** | 0.925* | -0.924* | 0.731 | 0.932* | 0.935* | 0.731 | -0.924* |
|  | 2,4-Decadienal, (E,E) | 0.974** | 0.920* | -0.919* | 0.717 | 0.927* | 0.931* | 0.717 | -0.919* |
|  | 2-Undecenal | 0.874 | 0.723 | -0.725 | 0.417 | 0.750 | 0.747 | 0.417 | -0.725 |
|  | GO | 0.986** | 0.928* | -0.940* | 0.748 | 0.954* | 0.944* | 0.748 | -0.940* |
|  | MGO | 0.985** | 0.923* | -0.920* | 0.726 | 0.930* | 0.935* | 0.726 | -0.920* |
|  | 2,3-BD | 0.949* | 0.950* | -0.967** | 0.883* | 0.976** | 0.961** | 0.883* | -0.967** |
|  | MDA | 0.992** | 0.965** | -0.963** | 0.814 | 0.968** | 0.973** | 0.814 | -0.963** |
|  | 4-HNE | 0.930* | 0.992** | -0.993** | 0.962* | 0.986** | 0.988** | 0.962* | -0.993** |

* Significance at P < 0.05. ** Significance at P < 0.01.

**Supplement Table 4.** Pearson correlation coefficients between the levels of oxidation products and fatty acids during heating in OO.

|  |  | C16:1 | C16:0 | C18:2 | C18:1 | C18:0 | SFA | MUFA | PUFA |
| --- | --- | --- | --- | --- | --- | --- | --- | --- | --- |
| OO | 2-Butenal | 0.869 | 0.860 | -0.976** | -0.950* | 0.993** | 0.890* | -0.953* | -0.976** |
|  | Pentanal | 0.789 | 0.823 | -0.969** | -0.901* | 0.976** | 0.855 | -0.906* | -0.969** |
|  | 2-Pentenal | 0.947* | 0.983** | -0.968** | -0.971** | 0.933* | 0.990** | -0.970** | -0.968** |
|  | Hexanal | 0.866 | 0.882* | -0.993** | -0.967** | 0.969** | 0.907* | -0.972** | -0.993** |
|  | 2-Hexenal | -0.966** | -0.969** | 0.966** | 0.975** | -0.968** | -0.982** | 0.973** | 0.966** |
|  | Heptanal | 0.856 | 0.869 | -0.991** | -0.959** | 0.979** | 0.896* | -0.963** | -0.991** |
|  | 2-Heptenal | 0.978** | 0.977** | -0.970** | -0.992** | 0.956* | 0.988** | -0.990** | -0.970** |
|  | 2,4-Heptadienal, (E,Z) | 0.938* | 0.944* | -0.907* | -0.963** | 0.827 | 0.942* | -0.962** | -0.907* |
|  | Octanal | 0.735 | 0.772 | -0.947* | -0.865 | 0.959** | 0.808 | -0.871 | -0.947* |
|  | 2,4-Heptadienal, (E,E) | 0.811 | 0.858 | -0.983** | -0.931* | 0.947* | 0.883* | -0.937* | -0.983** |
|  | 2-Octenal | 0.885* | 0.901* | -0.996** | -0.977** | 0.966** | 0.923* | -0.981** | -0.996** |
|  | Nonanal | 0.893* | 0.926* | -0.998** | -0.974** | 0.968** | 0.945* | -0.977** | -0.998** |
|  | 2-Decenal | 0.686 | 0.740 | -0.929* | -0.833 | 0.928* | 0.776 | -0.841 | -0.929* |
|  | 2,4-Decadienal, (E,Z) | 0.786 | 0.849 | -0.968** | -0.913* | 0.915* | 0.871 | -0.919* | -0.968** |
|  | 2,4-Decadienal, (E,E) | 0.759 | 0.826 | -0.959** | -0.892* | 0.914* | 0.850 | -0.899* | -0.959** |
|  | 2-Undecenal | 0.590 | 0.666 | -0.868 | -0.747 | 0.869 | 0.703 | -0.756 | -0.868 |
|  | GO | 0.884* | 0.874 | -0.981** | -0.964** | 0.987** | 0.901* | -0.967** | -0.981** |
|  | MGO | 0.929* | 0.916* | -0.933* | -0.977** | 0.877 | 0.924* | -0.978** | -0.933* |
|  | 2,3-BD | 0.911* | 0.904* | -0.937* | -0.973** | 0.874 | 0.914* | -0.975** | -0.937* |
|  | MDA | 0.790 | 0.804 | -0.962** | -0.900* | 0.983** | 0.840 | -0.906* | -0.962** |
|  | 4-HNE | 0.834 | 0.869 | -0.989** | -0.937* | 0.978** | 0.896* | -0.942* | -0.989** |

* Significance at P < 0.05. ** Significance at P < 0.01.

**Supplement Table 5.** Pearson correlation coefficients between the levels of oxidation products and fatty acids during heating in LO.

|  |  | C14:0 | C16:1 | C16:0 | C18:2 | C18:1 | C18:0 | SFA | MUFA | PUFA |
| --- | --- | --- | --- | --- | --- | --- | --- | --- | --- | --- |
| LO | 2-Butenal | 0.926* | -0.957* | 0.988** | -0.992** | -0.929* | 0.965** | 0.979** | -0.934* | -0.992** |
|  | Pentanal | 0.934* | -0.935* | 0.953* | -0.987** | -0.909* | 0.960** | 0.968** | -0.914* | -0.987** |
|  | 2-Pentenal | 0.962** | -0.958* | 0.969** | -0.995** | -0.973** | 0.995** | 0.997** | -0.976** | -0.995** |
|  | Hexanal | 0.814 | -0.83 | 0.921* | -0.926* | -0.857 | 0.890* | 0.902* | -0.859 | -0.926* |
|  | 2-Hexenal | 0.962** | -0.943* | 0.951* | -0.981** | -0.989** | 0.998** | 0.995** | -0.991** | -0.981** |
|  | Heptanal | 0.907* | -0.906* | 0.931* | -0.970** | -0.873 | 0.933* | 0.942* | -0.878 | -0.970** |
|  | 2-Heptenal | 0.971** | -0.951* | 0.949* | -0.981** | -0.989** | 0.999** | 0.996** | -0.991** | -0.981** |
|  | 2,4-Heptadienal, (E,Z) | 0.990** | -0.969** | 0.942* | -0.991** | -0.960** | 0.994** | 0.993** | -0.964** | -0.991** |
|  | Octanal | 0.766 | -0.762 | 0.833 | -0.871 | -0.745 | 0.815 | 0.826 | -0.75 | -0.871 |
|  | 2,4-Heptadienal, (E,E) | 0.986** | -0.970** | 0.949* | -0.994** | -0.955* | 0.993** | 0.993** | -0.960** | -0.994** |
|  | 2-Octenal | 0.959** | -0.934* | 0.945* | -0.982** | -0.982** | 0.996** | 0.991** | -0.984** | -0.982** |
|  | Nonanal | 0.915* | -0.912* | 0.949* | -0.979** | -0.914* | 0.956* | 0.962** | -0.918* | -0.979** |
|  | 2-Decenal | 0.938* | -0.937* | 0.930* | -0.978** | -0.872 | 0.942* | 0.950* | -0.879* | -0.978** |
|  | 2,4-Decadienal, (E,Z) | 0.935* | -0.938* | 0.944* | -0.984** | -0.890* | 0.951* | 0.960** | -0.896* | -0.984** |
|  | 2,4-Decadienal, (E,E) | 0.933* | -0.936* | 0.938* | -0.980** | -0.877 | 0.943* | 0.953* | -0.884* | -0.980** |
|  | 2-Undecenal | 0.912* | -0.918* | 0.931* | -0.970** | -0.859 | 0.928* | 0.939* | -0.866 | -0.970** |
|  | GO | 0.964** | -0.992** | 0.982** | -0.992** | -0.947* | 0.978** | 0.990** | -0.953* | -0.992** |
|  | MGO | 0.942* | -0.951* | 0.960** | -0.991** | -0.907* | 0.961** | 0.971** | -0.913* | -0.991** |
|  | 2,3-BD | 0.944* | -0.954* | 0.939* | -0.981** | -0.871 | 0.941* | 0.953* | -0.879* | -0.981** |
|  | MDA | 0.919* | -0.945* | 0.979** | -0.965** | -0.983** | 0.975** | 0.983** | -0.985** | -0.965** |
|  | HNE | 0.991** | -0.973** | 0.881* | -0.944* | -0.896* | 0.947* | 0.946* | -0.903* | -0.944* |

* Significance at P < 0.05. ** Significance at P < 0.01.


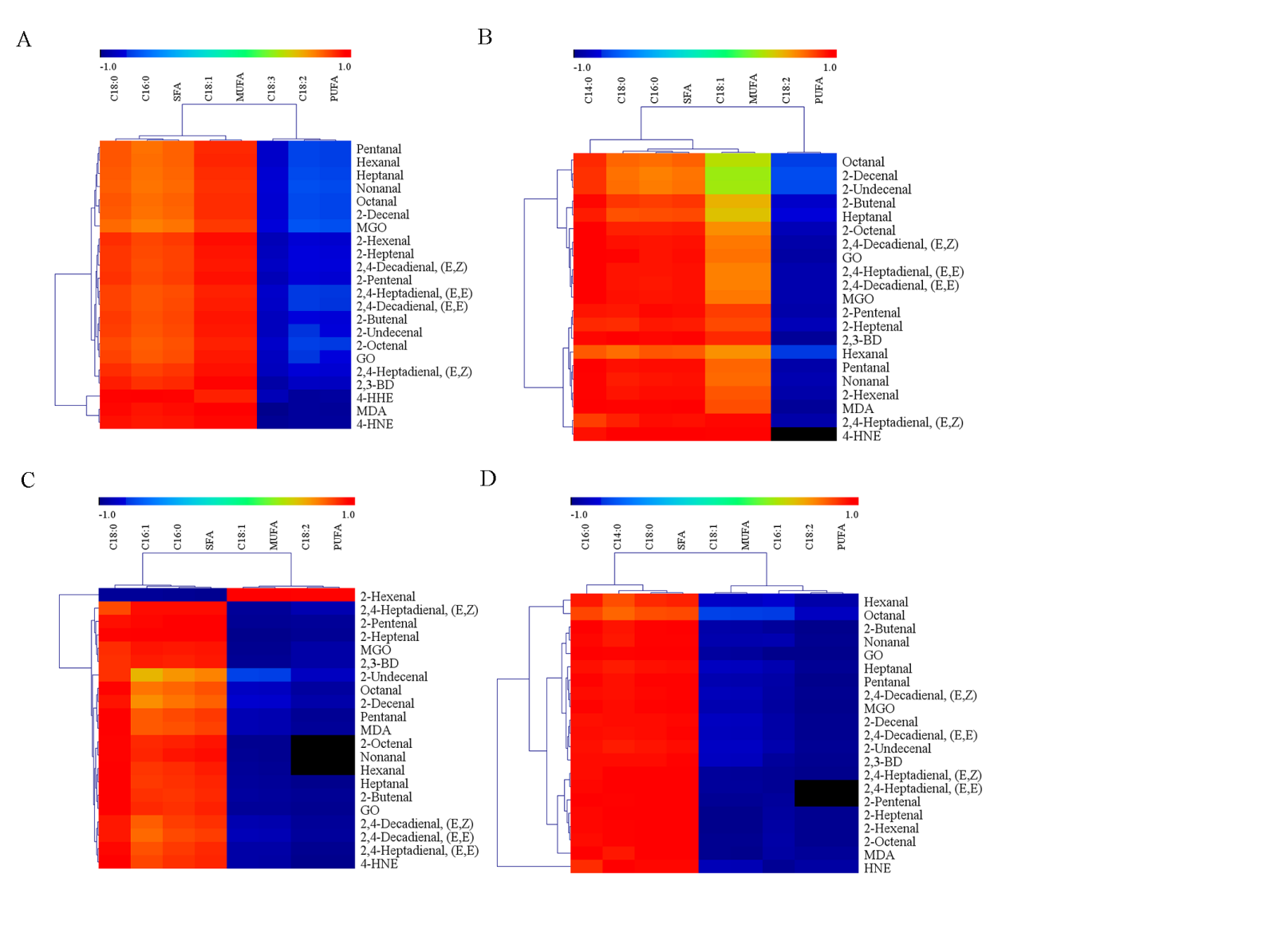
**Supplement Figure 1.**

Supplement Figure 1. Pearson’s correlation heatmap showing different indices in SO (A), PO (B), OO (C), and LO (D) at different temperatures.
